# Dr. Kinglake, on the Nature and Properties of Vital Power

**Published:** 1799-09

**Authors:** Robert Kinglake

**Affiliations:** Bristol


### *To the Editors of the Medical and Phyſical Journal.*

GENTLEMEN,

If you ſhould eſteem the following general obſervations on the *animal nature* and *properties of vital power*, deſerving a place in the department of your Journal appropriated to phyſiological ſpeculations, they are at your diſpoſal.

I am, Gentlemen,

Your's reſpectfully,

BRISTOL, *July* 17*th*, 1799. ROBERT KINGLAKE, M.D.

No inquiry has engaged the zeal of modern philoſophy with more ardour than the phenomena of vital power; and much ſubtility of reaſoning, and ingenuity of talent have been exerciſed on the ſubject, with various ſucceſs. The difficulties which nature preſents in this reſearch appear to be leſs formidable than thoſe which have ariſen from the errors of artificial inveſtigation. The real order of things is oftener overlooked, by allowing undue licenſe to efforts of imagination, than correctly perceived.

A notion of variety of power neceſſarily induces a correſpondent complexity in the opinion of its operation, and but too frequently eclipſes the more ſimple and intelligible courſe obſerved by the undeviating laws of nature.

To enumerate the various hypotheſes which have been, through every ſucceeding age, from remote antiquity to the preſent time, conceived and promulgated on this queſtion, would be at once unprofitably tedious, and inconſiſtent with ſuitable brevity; it is ſufficient to remark, that the labours of elapſed centuries on this topic have ſcarcely afforded information enough to ſerve as a rallying point to the modern diſtreſſes of ſpeculative deluſion. This diſaſtrous want of ſucceſs may be juſtly aſcribed to an intemperate deſire to explain, by precipitate generalization, what a cautious and ample accumulation of facts can alone develope.

Inſulated facts are not leſs uſeleſs than a deſultory and diſcordant concatenation tenation of them is obſtructive to the progreſſive improvement of ſcience. Truths, if ſuſceptible of legitimate union, require no adventitious aid to be ſyſtematized, but combine with natural and obvious facility; and when the connection does not ſpontaneouſly and evidently obtain, there is ſtrong reaſon to preſume, that ſome extraneous obſtacle, or deficient link, interrupts the continuity of the chain.

To avoid ſtumbling on the difficulties alluded to in the preceding obſervations, it is here only deſigned to ſuggeſt a few reflections on the apparently elementary nature of animal excitability.

This vital property is ſo inſeparably connected with the various conditions neceſſary to animal life, that it can no more be conſidered abſtractedly, than an effect can be intelligibly examined independently of its cauſe: it cannot, for the ſhorteſt duration, exiſt in a ſtate of diſorganization, in any manner cognizable to the human ſenſes; it muſt therefore be contemplated not as a cauſe, but as an effect reſulting from an uniform aſſemblage of operative laws, appoſitely preſcribed by phyſical neceſſity.

Now, as effects muſt neceſſarily aſſume and indicate the ſpecific character and diverſity of the cauſes from whence they originate, ſo in the various ſtructure and organization of the animal machine, is ſtrikingly exemplified a peculiar difference of excitability, accurately correſponding with the particular phyſical and chemical powers, reſpectively connected with ſuch diſſimilar conſtruction of parts.

Could the human eye be rendered ſufficiently microſcopic, to pry diſtinctly into the minuteſt integrant particles of animal organization, analogy derived from the more evolved ſtructure warrants the concluſion, that every organ would be found eſſentially different in the diſpoſition, form, number, and proportion of its radical and conſtituent principles; hence it is fair to infer, that the vital power manifeſted by the property of excitability, partakes of the nature and quality of ſuch diverſity; and conſequently it is allowable to affirm that the excitability of the brain is modified by the peculiar ſtructure of that organ, the ſame may be ſaid of all the thoracic and abdominal viſcera, likewiſe of the muſcular, nervous, vaſcular, cuticular, cellular, membranous, ligamentous, and oſſific arrangements of animal matter.

Although the property of excitability in theſe diſſimilar parts varies, as effects proceeding from different cauſes, yet ſimilarity in general principle and deſign joins and aſſociates every variety, in a ſpecies of indiviſible union, for the purpoſe of conſtituting and preſerving the integrity of the ſyſtem, and maintaining the vital and ſalutary relations of its various organs organs, hence similar general laws, modified by diversity of structure, govern and consolidate the animal frame, as a compound whole.*

If this view be correct, the notion of sensibility being an original and distinct property from the modified systematic excitability, is wholly untenable.

The property of sensibility results from a nervous modification of the general excitability, originates in the brain, and is distributed in suitable and relative diversity of structure† to every part of the system; in like manner, the digestive power, by which alimentary, and probably most medicinal substances,‡ are decomposed, and partly assimilated in the stomach, depends on the peculiar character of excitability, arising from the particular vital construction of that organ. Thus excitability may, with natural propriety, be denominated from the part in which it exists, such as cerebral, pulmonic, cardiac, diaphragmatic, stomachic, intestinal, hepatic, pancreatic, renal, &c. &c.§ An analogous unity of principle constitutes and governs by specific adaptation, the variously modified phenomena of corpuscular attraction and repulsion, the peculiar chemical affinities appertaining to the respective principles

---

* Diversæ corporis partes, suas rite præstantes functiones characterem vitæ humanæ certum ac distinctum faciunt ut vita totum pervadit mundum, sic forma ejus conspicua vim irritabilitatis afficit.

'*Dissertatio physiologica inauguralis de vitæ principio,*' &c.—Auct. Rob. Kinglake, 1794.

† Ich setze den specifischen unterschied der Nervenzeizbarheit in der verschiedenen structur der nerven. Es ist Thatsache, dass die nerven eine verschiedene structur haben.——'*Gesetze der Nerven reizbarheit,*' von Koellner.

‡ Parity of reason justifies the opinion, that medicinal substances, whose radical and constituent principles often resemble those of alimentary matter, both in general texture and chemical combination, should be equally subject to the decomposing and assimilating influence of the stomach, and that effects produced on remote parts of the system by such agents taken into the stomach, result from sympathetic propagation of impression made on that organ, and not from original or direct contact. It is further consonant with the provident security universally manifested in the animal economy, to suppose, that no description of matter could find admission into the sanguiferous system, by either the pulmonic, stomachic, or cuticular route, without previously undergoing a relative assimilation in those several situations; a secreting operation probably obtains, by which a suitable decomposition and recombination are produced.

§ Thus specifically nominating excitability tends usefully to direct the attention to a distinct physiological enquiry into the causes on which the peculiar vital properties of dissimilarly organized parts essentially depend.

ciples of light, electricity, magnetifm, and in fhort, the univerfal efficiency of matter.*

Thefe feveral powers are generated by the operation of phyfical and chemical forces, fpecifically adapted to produce thofe peculiar effects, and which, like vital organization, cannot exift without their refpective material arrangement.

The application of this principle is of the utmoft importance to the ftudy of every branch of phyfics, but to none more than that of phyfiology. It prefents the vital power in the character of an effect of material organization, and not as a caufe, and thus affords an elementary clue, by which to unravel the precife nature of the various agents that concur in producing, amidft an infinity of other wonderful phenomena, that moft fublime evolution—organic life. The agents by which the vital power is fupplied, fuftained, and perpetuated, are furnifhed by the various functions allotted to the feveral organs of the fyftem; the lungs, ftomach, brain, and cuticular furface, appear to be of the higheft importance in adminiftering to the inceffant wants of the animal economy.

How the exercife of thofe functions maintains the various phenomena of life; what is contributed by them to render the mufcular fibre contractile, and the nervous ftructure fentient; or in what ftationary or variable relation the power of being impreffed by acceffional agents confifts, is a queftion not incommenfurate with the progreffive elucidations of time and affiduity ultimately to folve, but at prefent involved in a degree of obfcurity widely difproportionate to the phyfiological means yet poffeffed for affording it a fatisfactory illuftration.

The agents which form the component parts of life are objects of fenfe, and capable of being fcrutinized in every way neceffary to arrive at the mode and effect of their operation.† It would undoubtedly be highly gratifying to fcience, to learn as much of the nature and character of vital power, as the antiphlogiftic doctrine of chemiftry has taught of the feparate and combined powers of fimple fubftances; to know, for example, as much of the

* Leges, vitæ principio, in omnibus rebus corporeis, existenti, impositas, sive in fabrica menti humanæ propria, vel in vegetabilium functionibus, vel in fossilium attractione, conspiciatur, philosopho recta ratione ducto investigare licitum est. Humana scientia hisce legibus accurate observatis excolatur multum et augeatur.

'*Diss. Phy. inaug. &c. auctore.*' R. Kinglake.

† Respirable air, light, and nutritious substances, are the most efficient vital agents.

the action of vital agents, as is underſtood of combuſtion, or of reſpiration, and the generation of animal heat by the direct effect of oxygen; but were this knowledge obtained, would one ſtep be gained toward the ſolution of the reaſon of ſuch a cauſe; or would the information go further than to verify that the vital power is inſeparably connected with certain agents, is a non-entity without their operative influence, and is liable to all the intermediate imperfection occurring between its full force and final extinction? Thus much is known of corpuſcular attraction, repulſion, gravity, and chemical affinity; but why ſuch effects ſhould obtain, can only be explained by referring to that decree which impoſes on matter the general and ſpecific laws of neceſſity.

Purſuant to the view propoſed of vital power in the preceding obſervations, the moſt philoſophical definition applicable to that principle, would appear to be, that it conſiſts in a peculiar attractive and repulſive force, ſubſiſting between the particles of animal matter, generated by the concurrent operation of various phyſical and chemical* agents; that, like the principle of light or electricity, it is ſuſceptible of general diſtribution, augmentation, diminution, and ſpecific modification, by which the laws of inanimate matter are reſiſted, and occaſionally aſſimilated to its own nature, in ſhort, that it exiſts as the neceſſary effect of a vital combination of attractive and repulſive motion.

---

### *To the Editors of the Medical and Phyſical Journal.*

Gentlemen,

SHOULD you think the following caſe of Hydrocephalus internus worthy of a place in your valuable periodical publication, by inſerting it in your next you will oblige,

Gentlemen,

Your very humble ſervant,

JAMES HUME SPRY,

London, July 16th 1799. *Surgeon, Alderſgate-ſtreet.*

Miſs

---

* Phyſical and chemical, in common with every diverſity of motive power, originate from the ſame univerſal principles of corpuſcularian attraction and repulsion, varying only in being generated by different arrangements of matter.

Much ingenious elucidation, and original application of the principles of corpuſcular attraction and repulsion, have been afforded by Mr. H. Davy's new Theory of Heat, Light, &c.

Vide '*Contributions to Phyſical and Medical Knowledge*,' collected by T. Beddoes, *M. D.*

